# A geometric assessment method for estimating dimensional change of retrieved dual mobility liners for total hip arthroplasty

**DOI:** 10.1177/09544119231176112

**Published:** 2023-05-24

**Authors:** Mackenzie Smeeton, Graham Isaac, Ruth Wilcox, James Anderson, Tim Board, Douglas W Van Citters, Sophie Williams

**Affiliations:** 1 Institute of Medical and Biological Engineering, University of Leeds, Leeds, UK; 2 DePuy Synthes Joint Reconstruction, Leeds, UK; 3 Wrightington, Wigan and Leigh NHS Foundation Trust, Wigan, UK; 4 Dartmouth College, Thayer School of Engineering, Hanover, NH, USA

**Keywords:** Total hip replacement, dual mobility, geometric assessment, coordinate measuring machine, explant analysis

## Abstract

Despite their emerging use, the current understanding of the in-vivo functional mechanisms of Dual Mobility (DM) Total Hip Replacements (THRs) is poor, and current characterisation methodologies are not suitable for the unique function and design of these types of devices. Therefore, the aim of this study was to develop a geometric characterisation methodology to estimate dimensional change across the articulating surfaces of retrieved DM polyethylene liners so that their invivo function may be better understood. The method involves the acquisition of three-dimensional coordinate data from the internal and external surfaces of DM liners. The data is processed using a bespoke MATLAB script which approximates the unworn reference geometry of each surface, calculates geometric variance at each point and produces surface deviation heatmaps so that areas of wear and/or deformation may be visualised across the implant. One as-manufactured and five retrieved DM liners were assessed, which demonstrated the efficacy, repeatability and sensitivity of the developed method. This study describes an automated and non-destructive approach for assessing retrieved DM liners of any size and from any manufacturer, which may be used in future research to improve our understanding of their in-vivo function and failure mechanisms.

## Introduction

Dual Mobility (DM) Total Hip Replacements (THRs) were introduced to overcome the challenges associated with joint dislocation, which is one of the most common indication for THR revision within the first 2 years following implantation.^1,2^ These implants are indicated to treat at-risk patients, such as those with neuromuscular conditions,^3^ abductor deficiencies,^4,5^ spinal fusions^6,7^ and skeletal cancers.^8^ Additionally, DM-THRs have been successfully used to treat fractures of the neck of femur.^9,10^ In contrast to unipolar THRs, DM constructs are characterised by an unconstrained polyethylene liner where both the internal and external surfaces are subject to articulation. This increases the effective head size of the implant thus improving stability and range of motion.

Good overall survivorship and low rates of dislocation have been reported in association with new generation DM-THRs.^11^ However, early evidence from the UK National Joint Registry suggest these bearings may have an increased revision rate within the first 5 years in comparison to unipolar bearings.^2^ Additionally, concerns about failure mechanisms unique to these implants, such as potential accelerated polyethylene wear at the three interfaces and intraprosthetic dislocation, remain a concern. Currently, the function and failure mechanisms of DM-THRs are not well understood. As these constructs are implanted more frequently and their use becomes more widespread, it is important to understand these mechanisms to improve implant design and long-term survivorship.

Implants retrieved during revision surgery can provide evidence on device function. Polyethylene liners from unipolar bearings have previously been assessed with various methodologies such as visual inspection,^12–14^ geometric assessment^15–17^ and microCT.^14,18,19^ Geometric assessment methodologies are particularly advantageous as they can provide information about the location, size, and shape of the surface changes such as damage and wear. In the context of DM-THRs, this could provide information about the in-vivo mechanics of the unconstrained polyethylene liners. However, previous attempts to geometrically assess retrieved DM liners have excluded large portions of the bearings surface and/or utilised sectioned (i.e. destructively-tested) components.^20,21^ Alternative approaches have successfully estimated volume loss but cannot provide information about the surface damage location or patterns due to a low number of sampling points.^22^ Therefore, the aim of this work was to develop a non-destructive geometric characterisation methodology for the semi-quantitative assessment of the articulating surfaces of DM polyethylene liners for wear and/or deformation. The repeatability of the method was assessed, and several retrieved and as-manufactured liners were measured to verify that the method could detect changes to the surface geometry of these components.

## Methods and materials

### Measurement method

A Legex 322 coordinate measuring machine (Mitutoyo, UK) was programmed to capture three-dimensional geometric data from the articulating surfaces of DM liners. The machine was configured with a single straight stylus with a 6-mm head diameter.

**Figure 1.**
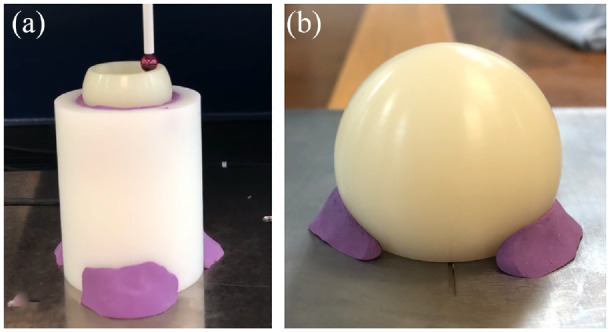
Liners were securely fixed to the measurement bed of the CMM using bespoke fixtures to facilitate measurements of the internal (a) and external (b) surfaces. Small amounts of plasticine were introduced at the outer edges of the components to prevent surface damage and component rotation.

Liners were securely fixed as shown in Figure 1 and measured through a series of 144 traces spaced in 2.5° intervals about the vertical axis. Each trace originated from the pole and terminated at either the retentive bore (for internal surface measurements) or 3-mm beyond the equator (for external surface measurements). The measurement time and number of acquired data points was dependent on the size of the sample. In the case of a BI-MENTUM™ liner (DePuy Synthes) with an inner diameter of 28-mm and outer diameter of 63-mm, which represents the largest available DM liner size available, the measurement time was 93 min which resulted in the acquisition of approximately 15,000 internal and 26,000 external coordinate points.

The coordinate data was analysed using a bespoke MATLAB script (Version 2020b, MathWorks). Insufficient pre-service information is available for retrieved implants and thus the unworn reference geometry was approximated with a sphere fitting algorithm. This approach was considered desirable in comparison to alternative methods such as utilising manufacturer-supplied CAD models or part drawings, as these do not consider individual variations between components due to manufacturing tolerances.

The geometric variation of each point was determined as the radial distance to the reference geometry. Positive geometric variation denotes penetration into the surface whilst negative geometric variation is representative of protrusion out of the reference geometry. Geometric variance may be caused by either wear (i.e. material loss) or deformation, and the method cannot distinguish between the two.

**Figure 2.**
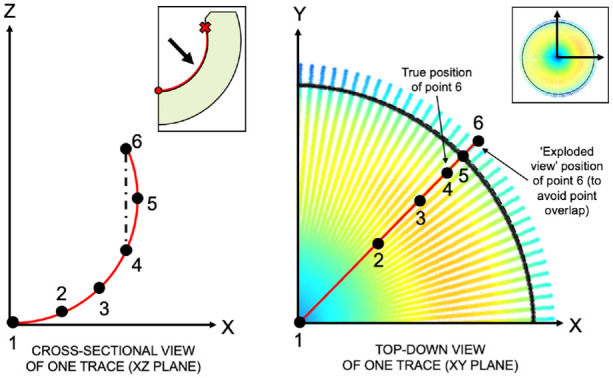
A cross-sectional view (XZ-plane) of one measurement trace is shown on the left, which features six example points. Due to the supra-hemispheric geometry of DM liners, Points 4 and 6 would overlap on a top-down (i.e. XY-plane) geometric variance heatmap of the surface. Therefore, points situated beyond the equator were represented in an ‘exploded’ form as shown by the image on the right to ensure the entire surface could be visualised. The location of the equator is marked by a black circle to make the distinction between the lower and upper hemispheres clear.

To visualise areas of surface damage, the coordinate data was plotted, and each point was assigned a colour based on its geometric variance. To ensure full surface visualisation, the spherical surface above the equator was displayed in an exploded view as described in Figure 2.

**Figure 3.**
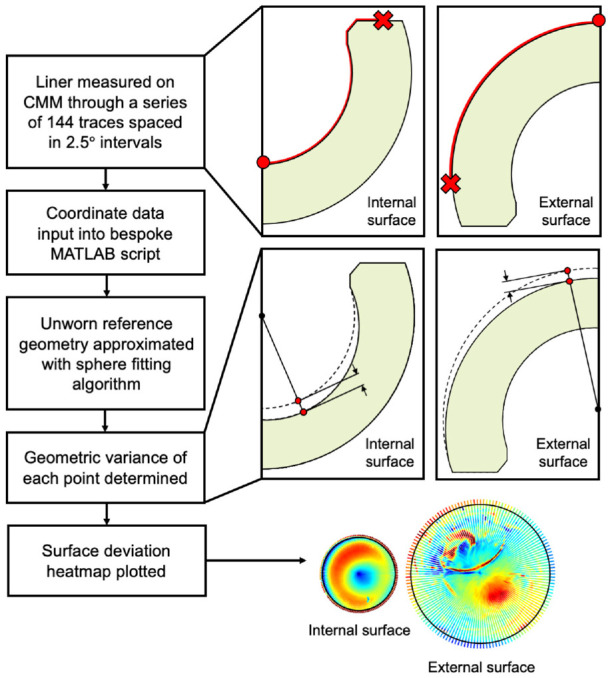
Flowchart describing the geometric assessment protocol.

A summary of the measurement methodology is depicted in Figure 3.

### Repeatability analysis

To assess the repeatability of the method, the surfaces of one in-vitro tested BI-MENTUM™ liner (DePuy Synthes, UK), featuring a 28-mm internal and 63-mm external diameter, were measured on five separate occasions. The liner was oriented in the same position for the first three measurements, and then rotated by 90° and 180° for the final two repeats. Several outputs were recorded which included the radius of the unworn reference sphere and geometric variance (mean, median, minimum, maximum) of each surface. Surface deviation heatmaps were also qualitatively assessed.

### Method verification

Five retrieved DM polyethylene liners were assessed to verify the methodology was able to detect changes to the surface geometry and accurately represent damaged features found on their surfaces. The liners were sourced from retrievals collection programs which obtained ethical approval from the following review boards: IRB CPHS at Dartmouth College, USA (reference: STUDY00022199) and NRES at Greater Manchester West, UK (reference: 18/NW/0707). Implants were selected based on the presence of damaged or gouged regions which were visible by eye. The damaged features were identified only on the outer bearing surface of the polyethylene liners. The surface deviation heatmaps were inspected for the presence of these damaged regions which were visually compared to the samples. Five as-manufactured DM liners (BI-MENTUM™ liner with a 28-mm internal and 63-mm external diameter; DePuy Synthes) were also assessed as a control.

## Results

The data associated with this paper are openly available from the University of Leeds Data Repository.^23^

### Repeatability analysis

The method was shown to repeatably approximate the unworn reference geometry over five repeated trials, whereby the reference radius varied by a standard deviation of ±2 µm and ±1 µm for the internal and external surfaces over the five repeated trials, respectively. Additionally, the geometric variance (mean, median, maximum and minimum) varied by a standard deviation of ±2 µm across all repeats. No qualitative differences were observed in the surface deviation heatmaps between the five repeats.

### Method verification

**Figure 4.**
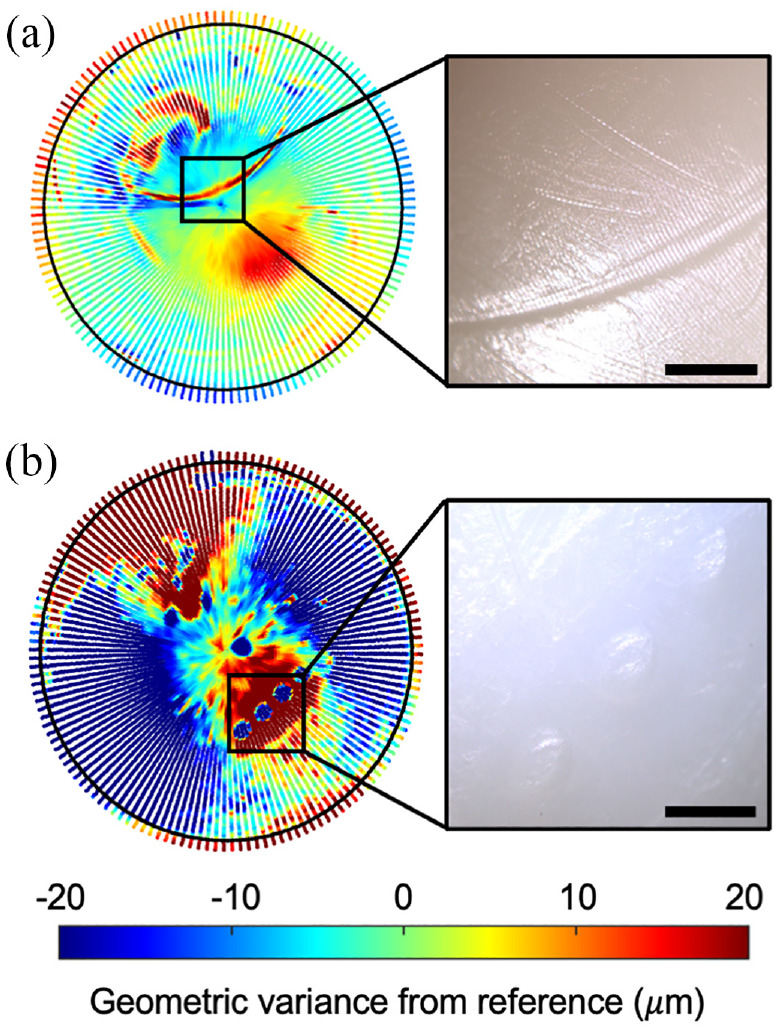
External surface deviation heatmaps of two retrieved DM liners, and corresponding microscope images (a) Sample 15; (b) Sample 19. Scalebars represent a unit of 2 mm.

**Figure 5.**
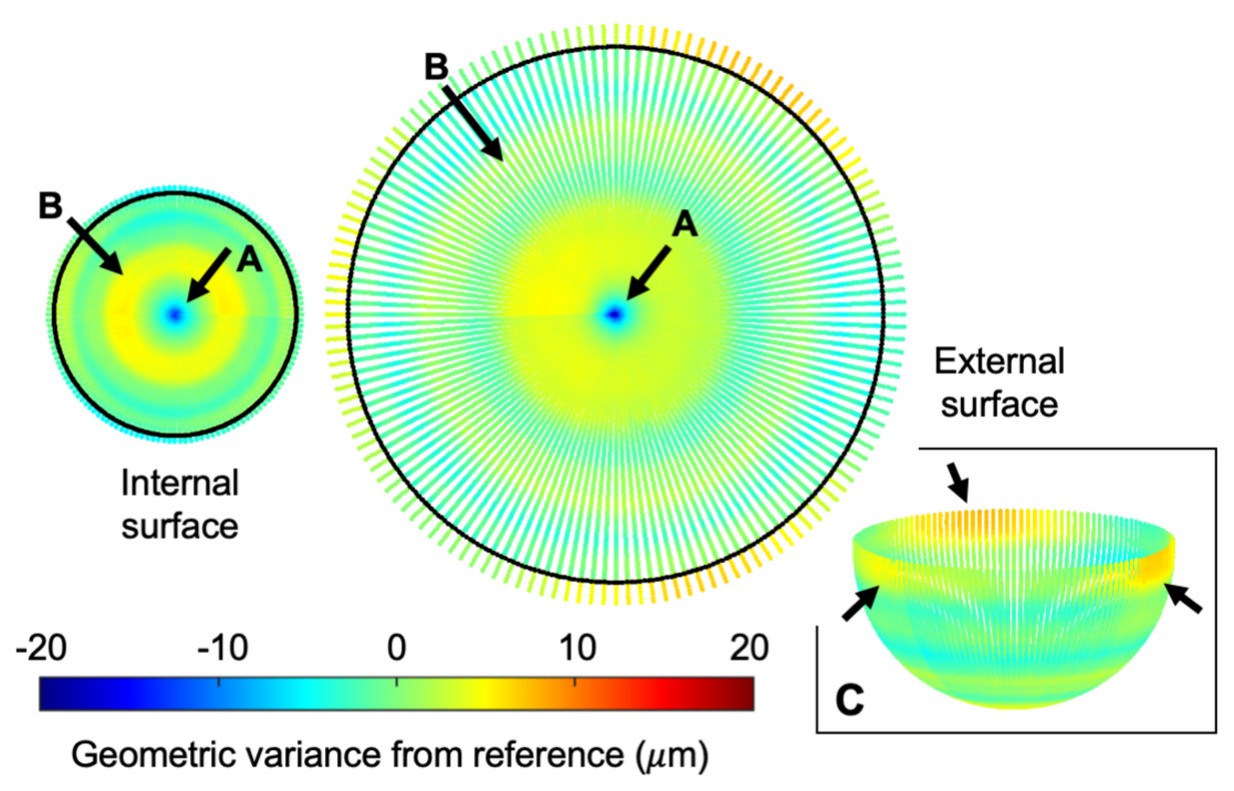
Surface deviation heatmap of an as-manufactured DM liner, depicting a small region of protruding material at the pole (A) and a circumferential stripe (B) on each of the surfaces. Three penetrating regions were also noted on the upper hemisphere of the external surface (C).

Damaged features observed visually on the outer surfaces of retrieved DM liners were clearly identified on the surface deviation heatmaps as exampled in Figure 4. The damaged features could be described as regions of deep penetrating gouges (*n* = 3 liners), a cluster of four protruding circular marks (*n* = 1 liner) and a C-shaped indentation (*n* = 1 liner). As expected, the as-manufactured samples showed no visible signs of surface damage as demonstrated in Figure 5. Artefacts of the manufacturing process include circumferential machining marks and a small region of protruding material (<20 µm in height) at the pole of each surface. Additionally, three penetrating regions were identified on the spherical surface above the equator of the external surfaces.

## Discussion

At present, there is a poor understanding relating to the in-vivo function and failure mechanisms of DM-THRs. There is evidence to suggest that DM-THRs may be susceptible to higher failure rates than unipolar bearings, although it remains unclear whether this is directly related to the design and performance of DM devices or, instead, an artefact of the inherent differences in demographics and pre-existing comorbidities which may exist between patients treated with unipolar and DM implants. Therefore, enhanced monitoring of DM-THRs is essential particularly considering their emerging use within both elective and trauma orthopaedic settings.

This study has demonstrated the successful development of a geometric characterisation methodology for the assessment of the articulating surfaces of DM polyethylene liners for dimensional change. The method produces surface deviation heatmaps through a novel, two-dimensional representation of the coordinate data which allows the full bearing surface to be visualised despite its supra-hemispheric geometry. This may be used in future studies, such as retrieval analyses or experimental simulations, to assess the surface damage, in-vivo function and pertinent failure mechanisms of these types of devices. This information has the potential to aid in the development of next-generation implant designs, and to provide surgeons with more informed operative guidelines relating to the optimal and worst-case use of DM-THRs.

The method benefits from a fully automated measurement protocol which does not require the use of sectioned components or exclusions of large regions of the articulating surface, unlike previous attempts to geometrically assess DM liners as reported in the literature.^20,21^ In the described method, only a thin band near the rim of the external surface was excluded due to limitations of the equipment setup thus allowing damage across the majority of the articulating surfaces to be assessed.

The method is compatible for use with both retrieved and in-vitro tested DM liners from any manufacturer or of any size. This is due to its data analysis protocol, which relies on the use of a sphere fitting algorithm to approximate the unworn reference geometry of each implant individually. This is advantageous to alternative approaches previously explored in the literature, such as utilising data from pristine components^20^ or manufacturer-supplied CAD models or component drawings,^21^ as these do not consider the manufacturing variations which exist between implants. Ultimately, this increases the utility of the method as researchers do not require access to pristine components or commercially-sensitive data to undertake this type of assessment.

The sensitivity of the method was demonstrated through the analysis of an as-manufactured sample, whereby manufacturing artefacts with geometric variances below 20 µm were able to be detected. Additionally, the method was shown to be repeatable and robust against variations in component orientation.

The method is limited by its inability to distinguish between wear and deformation of the sample. This is a recognised limitation of geometric methods to assess THR liner damage. In addition, the described method does not consider damage at the retentive bore or chamfer of the samples. This is a region of interest for DM components, as degradation of this region may lead to a serious complication known as intraprosthetic dislocation, and thus should be considered in future investigations.
